# Multifunctional Silk Fibroin Hydrogel with Antibacterial and Regenerative Properties for Accelerated Wound Healing

**DOI:** 10.3390/gels12050417

**Published:** 2026-05-10

**Authors:** Yanjiao Wu, Jiayue Chen, Luyao Han, Yiqiong Zhang, Li Wei

**Affiliations:** School of Pharmacy, Shanghai University of Traditional Chinese Medicine, Shanghai 201203, China; m17839976445@163.com (Y.W.); cjybaby0815@163.com (J.C.); hanluyao028@163.com (L.H.); zyq20210828@163.com (Y.Z.)

**Keywords:** total saponins of *Panax notoginseng* flower buds, silk fibroin, dual-dynamically crosslinked hydrogel, antibacterial, multifunctional wound dressings

## Abstract

The emergence of multifunctional wound dressings represents a significant transformation in the care of cutaneous tissue injuries, providing advanced solutions that surpass traditional dressings. This study is poised to fabricate multifunctional hydrogels through dual-dynamic cross-linking, integrating antibacterial and antioxidant properties, which are capable of accelerating wound healing while improving therapeutic outcomes. The hydrogel, which exhibits excellent adhesion, rapid self-healing ability, and on-demand removability, was synthesized employing poly(vinyl alcohol) (PVA)–borax as the backbone, followed by the incorporation of silk fibroin (SF), tannic acid (TA), and chitosan (CS). Total saponins of *Panax notoginseng* flower buds (PNF) with anti-inflammatory and angiogenic properties were loaded in porous structural materials yielding the PBCTS@PNF hydrogel. The prepared hydrogel exhibited outstanding antioxidant properties and cytocompatibility, along with favorable antibacterial capabilities, achieving inhibition rates of 84.30 ± 2.34% against *Escherichia coli* (*E. coli*) and 98.12 ± 0.76% against *Staphylococcus aureus* (*S. aureus*), respectively. Animal experiments demonstrated that PBCTS@PNF significantly reduced inflammation and promoted multidimensional tissue regeneration, encompassing re-epithelialization, neovascularization, and hair follicle regeneration, along with ordered collagen matrix organization, leading to substantially accelerated wound healing. The multifunctional PBCTS@PNF hydrogel provides a potent bioengineered therapeutic platform for wound healing management through the synergistic interplay among antibacterial, anti-inflammatory, and tissue regenerative functionalities.

## 1. Introduction

The skin, serving as the body’s foremost physical and immunological barrier, plays a pivotal role in resisting microorganisms, regulating metabolism, and maintaining internal homeostasis [1,2]. However, prolonged exposure to the external environment renders the skin vulnerable to exogenous insults or endogenous disorders, leading to acute or chronic wounds of diverse dimensions and depths that compromise patients’ daily function and quality of life [3]. Wound healing is a continuous and intricate process divided into four sequential phases: hemostasis, inflammation, proliferation, and remodeling [4]. Wounds are susceptible to microbial infection, which may subsequently trigger a range of serious systemic disorders, including metabolic derangements [5,6]. Thus, selecting an appropriate dressing is crucial for wound healing.

While conventional medical materials often lack the capacity to actively accelerate wound healing, emerging alternatives show a promising prospect. Hydrogel is regarded as an ideal wound-dressing material due to its three-dimensionally cross-linked architecture, which provides a soft consistency, excellent biocompatibility, and an inherent porous network. Through judicious selection of precursor components, a broad spectrum of functionalities can be incorporated. Moreover, the versatile design of hydrogel systems enables diverse biomedical functionalities, including antimicrobial, antioxidant, and growth factor-mimetic properties, which provides a versatile platform for wound repair. To satisfy the clinical needs of advanced wound management, hydrogel dressings must be designed with multifunctional capabilities. Firstly, an ideal dressing should exhibit excellent biocompatibility to prevent adverse immune responses while maintaining a consistent moist environment essential for re-epithelialization. Secondly, given the dynamic nature of wound sites, the material should possess robust mechanical resilience and rapid self-healing capacity to withstand repeated deformation and recover structural integrity following disruptive insults. Moreover, balanced tissue adhesion is critical: sufficient adhesion ensures dressing fixation and prevents bacterial infiltration, yet excessive adhesion risks traumatic removal and secondary injury to regenerating tissue upon dressing change. Particularly during the inflammatory phase, wounds produce high levels of reactive oxygen species (ROS) and are highly susceptible to bacterial infection, which severely impairs tissue regeneration and repair efficiency [7]. Therefore, the development of multifunctional hydrogels represents a necessary evolution from traditional single-function dressings, directly addressing the clinical demand for personalized, intelligent, and integrated wound management.

Silk fibroin (SF) is a natural polymer that has received approval from the U.S. Food and Drug Administration (FDA) for specific medical applications. Owing to its excellent biocompatibility and pro-healing capacity, silk fibroin is considered an ideal substrate for the preparation of wound dressings [8]. However, pristine silk fibroin hydrogels exhibit notable deficiencies in tissue adhesion and antibacterial activity, making it difficult to independently meet the comprehensive requirements of advanced wound care. Therefore, multifunctional modification of silk fibroin has become a key strategy to enhance its clinical applicability. A promising approach is the incorporation of tannic acid (TA). The unique molecular structure of TA enables strong interactions with proteins and enhances adhesion to biological tissues. Moreover, TA possesses both antibacterial and antioxidant functions, further improving its therapeutic efficacy [9]. It has been demonstrated that increasing the tannic acid content markedly enhances both the adhesive performance and antibacterial activity of silk fibroin hydrogels [10]. Meanwhile, the introduction of chitosan (CS) has been found to act synergistically with TA in exerting antibacterial activity, as confirmed by multiple antibacterial studies [11,12]. By integrating a dynamic crosslinking strategy (PVA–borax network), the mechanical resilience and reusability of the material are further enhanced, making it suitable for applications that require multiple cycles of adhesion and detachment.

In recent years, a growing body of research has highlighted the distinctive advantages of Traditional Chinese Medicine (TCM) in wound care [13,14,15,16]. TCM exerts multi-component, multi-pathway and multi-target synergistic effects, and plays an important role in promoting wound healing by modulating relevant signaling pathways, inhibiting pro-inflammatory cytokine release, reducing oxidative stress and promoting collagen synthesis [17]. The integration of TCM with hydrogel-based drug delivery systems not only overcomes inherent physicochemical limitations, such as poor aqueous solubility, chemical instability, and uncontrolled release kinetics, but also elevates TCM application to an engineered, precision-oriented modern therapeutic paradigm through stimuli-responsive functionalities and microenvironmental modulation [18,19,20,21]. Total saponins of *Panax notoginseng* flower buds (PNF) are derived from the dried flower buds of *Panax notoginseng* (Burk.) F.H. Chen. Modern medical research has demonstrated that PNF exhibits beneficial effects including anti-inflammatory, analgesic, hypotensive, and sedative actions, as well as the enhancement of human immune function [22]. Specifically, our prior work demonstrated that total saponins from PNF exert significantly stronger pro-angiogenic and vascular-protective effects than their root-derived counterparts [23]. Furthermore, we developed a PNF-based hydrogel capable of attenuating infection while promoting granulation tissue formation and epidermal hyperplasia [24].

Building upon these foundations, a multifunctional hydrogel (PBCTS@PNF) with antibacterial, antioxidant, and wound healing-promoting capabilities was designed in this study. This hydrogel employs silk fibroin as the matrix, with poly(vinyl alcohol) (PVA)–borax complex serving as the structural scaffold. The introduction of TA and chitosan (CS) enables functionalization of the silk fibroin hydrogel through hydrogen bonding, π-π stacking, and hydrophobic interactions with silk fibroin. The reversible dynamic borate ester bonds formed between PVA and borax act synergistically with the aforementioned non-covalent interactions, endowing the hydrogel with self-healing ability, wet adhesion, and pH responsiveness. TA provides antioxidant and anti-inflammatory effects, while the TA-CS complex exhibits synergistic antibacterial activity. The incorporated PNF exhibits anti-inflammatory properties [25] and promotes cell proliferation, migration, and angiogenesis [26,27]. Collectively, these multifunctional components act in concert to accelerate wound healing. To evaluate the integrated performance of this system, we conducted systematic studies encompassing the rational design, fabrication, and comprehensive characterization of the PBCTS@PNF hydrogel, including physicochemical properties (porosity, swelling, adhesion, rheology, self-healing, and pH/sugar responsiveness), in vitro performance (cytocompatibility, sustained drug release, antioxidant, and antibacterial activities), and in vivo efficacy in a full-thickness wound model. Overall, owing to its highly adaptable performance and comprehensive therapeutic efficacy, the PBCTS@PNF hydrogel offers a promising strategy for advanced wound management and represents a prospective candidate in the field of tissue regeneration.

## 2. Results and Discussion

### 2.1. Design and Preparation of the Hydrogels

**Figure 1:**
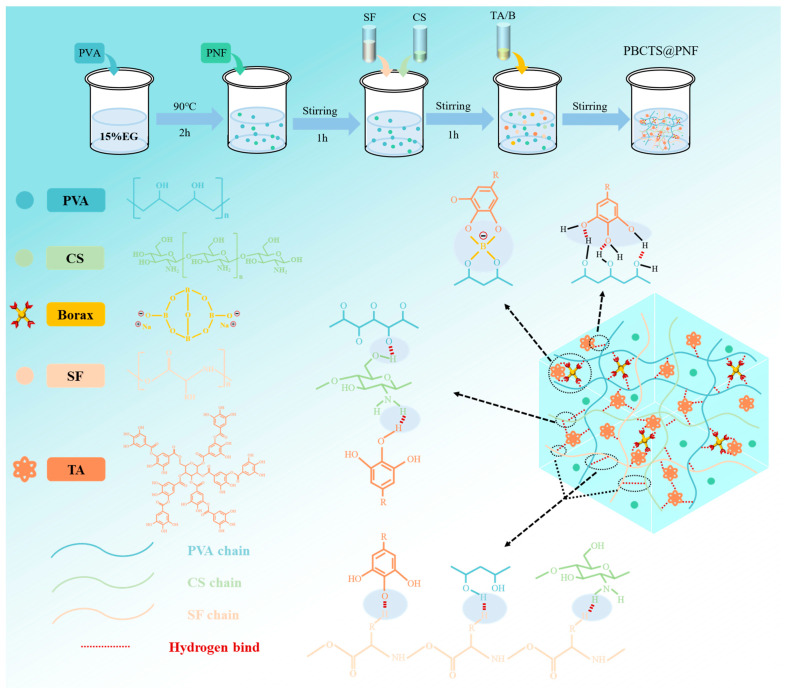
Schematic diagram of the hydrogel preparation process and gelation mechanism.

As illustrated in Figure 1, the PBCTS hydrogel forms a three-dimensional, multi-level crosslinked network. Natural SF offers excellent biocompatibility, mechanical properties, and wound healing promotion, making it an ideal wound dressing base material. Currently, most SF hydrogels rely on β-sheet self-assembly or chemical modification. However, β-sheet self-assembly leads to poor mechanical properties, adhesion, and reproducibility, while chemical modification introduces residual crosslinking agents, limited secondary modification options, and delayed degradation. To overcome these limitations, we physically blended SF into the hydrogel network, eliminating the need for chemical crosslinking agents and yielding a novel dressing with tailored mechanical properties, suitable adhesion, biocompatibility, and rapid self-healing. Accordingly, we selected the PVA–borax system as the hydrogel backbone. Borax rapidly dissolves in water to generate B(OH)_3_ and B(OH)_4_^−^, which form reversible borate ester bonds with the cis-diol groups on PVA chains, conferring rapid self-healing. TA, rich in catechol groups, was introduced to enhance tissue adhesion by forming hydrogen bonds, ionic interactions, and π-π stacking with SF’s peptide bonds, hydroxyl groups, and amino groups, creating a dynamic polyphenol–protein network that endows mussel-inspired rapid adhesion and self-healing. These two dynamic bond types (borate esters and hydrogen bonds) impart superior mechanical performance and structural stability, and can quickly reform after damage to restore original properties. The PBCTS network primarily consists of reversible borate ester bonds (between PVA cis-diols and B(OH)_4_^−^) and hydrogen bonds (between TA and SF). Additionally, CS with broad-spectrum antibacterial properties was incorporated to synergistically enhance antimicrobial activity with TA, facilitating wound healing application. Finally, freeze–thaw cycles render the dynamic crosslinked network more regular and stable, further enhancing mechanical performance.

### 2.2. Characterization of the PBCTS Hydrogels

#### 2.2.1. Structural Analysis

**Figure 2:**
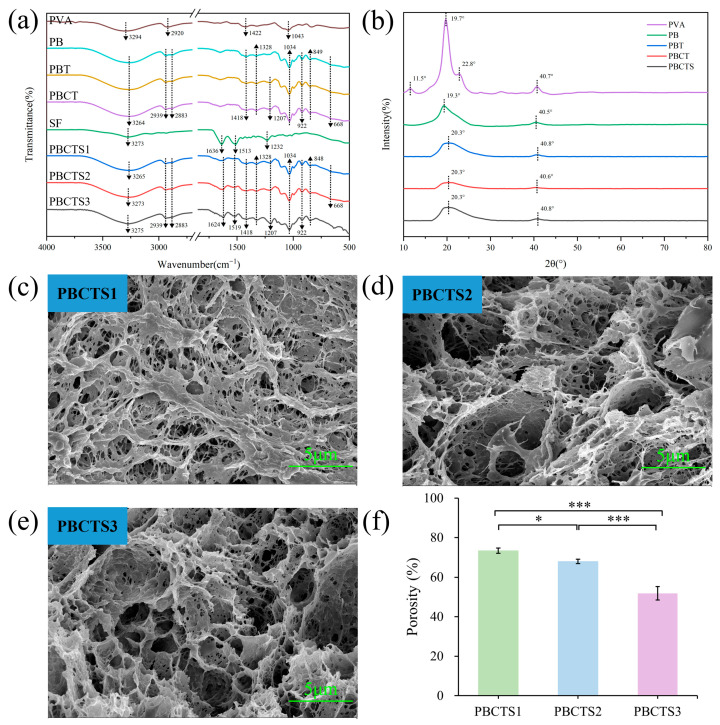
The structural analysis of PBCTS hydrogels. (**a**) FTIR spectra. (**b**) XRD patterns. The SEM image of PBCTS1 hydrogel (**c**), PBCTS2 hydrogel (**d**), and PBCTS3 hydrogel (**e**). (**f**) Porosities of PBCTS1, PBCTS2, and PBCTS3 hydrogels. * *p* < 0.05, *** *p* < 0.001.

Fourier transform infrared (FTIR) spectroscopy was employed to elucidate the cross-linking mechanism and chemical structure of PBCTS hydrogels (Figure 2a). In the neat PVA spectrum, characteristic peaks at 2920 cm^−1^, 1422 cm^−1^ and 1043 cm^−1^ arise from C–H stretching/bending of methylene groups and C–O stretching of primary alcohols [28,29]. Upon borax incorporation, all hydrogel spectra display characteristic peaks of borate ester bonds and borate anions. Specifically, peaks at 1418 cm^−1^ and 1328 cm^−1^ correspond to antisymmetric C–O–B stretching, indicating tetrahedral and trigonal borate complexes [30,31], while peaks at 849 cm^−1^ and 668 cm^−1^ arise from B–O stretching of residual B(OH)_4_^−^ and B–O–B bending [32]. These peaks confirm that borax reacts with PVA to form borate ester bonds, with a small amount of borate anions remaining in the network. The persistence of the 1418 cm^−1^ and 1328 cm^−1^ peaks in PBT (PVA–borax–TA), PBCT (PVA–borax–CS–TA), and PBCTS (PVA–borax–CS–TA–SF) spectra demonstrates that TA, CS, and SF do not interfere with borate ester bond formation. TA incorporation introduces an absorption band at 1207 cm^−1^ (phenolic C–O stretching), and the decreased intensity of the 3264 cm^−1^ peak—attributed to reduced hydroxyl vibrational freedom from additional hydrogen bonds—indicates good TA integration with other components. Compared to neat SF, slight shifts in PBCTS amide I (1636 cm^−1^), II (1513 cm^−1^), and III (1232 cm^−1^) bands [33,34] provide evidence that SF primarily interacts with other components via hydrogen bonding during gelation, accompanied by a conformational transition of SF from random coil to β-sheet. Collectively, these results confirm the successful construction of a three-dimensional crosslinked PBCTS network through borate ester bonds and hydrogen bonds among all components.

The crystalline structure of the hydrogels was investigated using X-ray diffraction (XRD) (Figure 2b). Neat PVA shows characteristic diffraction peaks at 2θ = 11.5°, 19.7°, 22.8°, and 40.7° [35,36]. Upon borax addition, the sharp peaks at 19.7° and 40.7° are significantly attenuated and broadened, while the peaks at 11.5° and 22.8° disappear, indicating that strong interactions between borax and PVA prevent PVA chain aggregation and disrupt its crystalline structure [37]. Subsequent incorporation of TA, CS, and SF into the PB hydrogel causes no significant changes in the diffraction peaks of PBT, PBCT, and PBCTS, with only slight variations in peak position and intensity attributable to additional hydrogen bonds between the newly added components and PVA.

Scanning electron microscopy (SEM) was used to characterize the microstructure of PBCTS hydrogels with varying SF contents (Figure 2c–e). All lyophilized hydrogels exhibit interconnected three-dimensional porous networks. As SF content increases, the pores become smaller and more uniform. PBCTS2 and PBCTS3 hydrogels not only possess uniform and dense pores but also display distinct micro- and nano-scale roughness on the pore walls, which facilitates drug loading and provides an excellent physical microenvironment for cell adhesion and growth. This rough porous structure is crucial for achieving the dual functions of drug delivery and tissue regeneration.

Porosity was analyzed using the liquid displacement method (Figure 2f). All three hydrogels exhibited porosity exceeding 50%, forming a three-dimensional interconnected porous structure favorable for mechanical properties and drug delivery [38]. Porosity decreases with increasing SF content, likely due to increased crosslinking density among components. Additionally, un-crosslinked SF undergoes a conformational transition from random coil to β-sheet during freeze–thaw cycles, thereby forming a porous architecture and reinforcing crosslinking between molecular chains.

#### 2.2.2. Water Retention Properties

**Figure 3:**
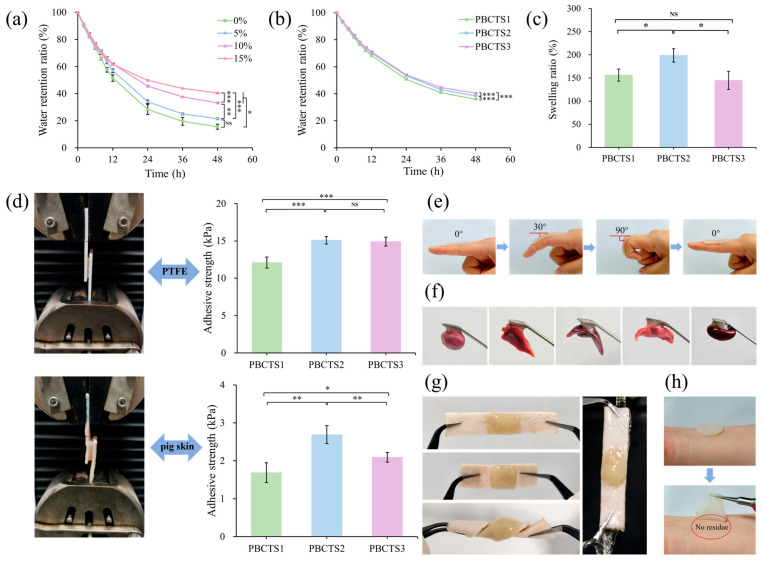
(**a**) Water retention ratio of hydrogel with different amounts of glycerol. (**b**) Water retention ratio of hydrogel with different amounts of SF. (**c**) Swelling ratio of PBCTS1, PBCTS2, and PBCTS3. (**d**) Adhesive strength by pig skin and PTFE. (**e**) Skin conformability. Adhesion of hydrogel to visceral organs (**f**) and porcine skin (**g**). (**h**) On-demand removability without residue. NS *p* > 0.05, * *p* < 0.05, ** *p* < 0.01, *** *p* < 0.001.

A moist healing environment promotes wound healing and protects against bacterial infection [39,40]. However, prolonged air exposure causes hydrogels to lose water, leading to deterioration of adhesion, elasticity, and flexibility, which compromises their application in wound healing. Therefore, excellent moisture retention is particularly crucial for hydrogel wound dressings. As shown in Figure 3a, the hydrogel without glycerol retained only 15.61% moisture after 48 h, exhibiting pronounced shrinkage and desiccation. In contrast, glycerol-containing hydrogels showed substantially improved moisture retention, with increasing glycerol content progressively suppressing water evaporation; 15% glycerol yielded the most pronounced reduction in evaporative loss. As illustrated in Figure 3b, hydrogels with different SF contents exhibit nearly identical water evaporation trends that scale inversely with porosity, indicating a unified mechanism: denser crosslinking and smaller pores retard water loss.

#### 2.2.3. Swelling Properties

Swelling capacity is a fundamental physicochemical property of hydrogels, directly dictating their performance in drug delivery, tissue engineering, and sensor applications [41]. Hydrogel swelling is governed by multiple factors, with the density of hydrophilic moieties and crosslinks directly determining the elastic retractive force of the network, serving as key regulators of swelling capacity. Figure 3c presents the equilibrium swelling ratios of PBCTS hydrogels with varying SF contents. As SF content increases, the equilibrium swelling ratio initially rises and then declines. This behavior is primarily attributed to the amphiphilic nature of SF. At an optimal SF dosage, abundant hydroxyl, carboxyl, and amino groups establish extensive hydrogen bonding with water, enhancing water uptake, while the simultaneously increased crosslinking density reinforces the network, allowing the hydrogel to absorb more water without rupturing. However, excessive SF promotes the formation of β-sheet crystallites via hydrophobic stacking, which act as physical crosslinks. These crystallites introduce a secondary physical network superimposed on the chemical network, markedly increasing overall crosslinking density and reducing porosity. The denser structure impedes water diffusion into the hydrogel, leading to a pronounced decrease in the equilibrium swelling ratio [42,43].

#### 2.2.4. Adhesive Properties

Optimal adhesive strength enables instantaneous wound sealing, creating a physical barrier against bacterial invasion while preserving mechanical congruence between the hydrogel and native tissue [44,45]. This stable interface suppresses micro-motion and fluid infiltration, providing a permissive microenvironment for angiogenesis and re-epithelialization that accelerates wound closure [46,47]. Adhesive performance was characterized by lap-shear testing using porcine skin and polytetrafluoroethylene (PTFE) as substrates (Figure 3d). PBCTS exhibits excellent adhesion to both substrates, as TA and SF are intrinsically adhesive, and the mussel-inspired moieties generated by the reaction between their catechol hydroxyls further synergistically endow the network with outstanding interfacial binding capacity [48,49]. Notably, the adhesive strength difference between the two substrates is marginal, with a slightly higher value observed on PTFE. Adhesive strength toward both substrates peaked at 3.0% SF content, attributed to optimal crosslinking density and stiffness at this concentration. This PBCTS2 hydrogel was selected for further macroscopic adhesion evaluation. It exhibits excellent skin conformability (Figure 3e) and robust adhesion to various soft biological tissues, including heart, liver, spleen, lung, kidney, human skin, and porcine skin tissues (Figure 3f–h). When applied to human skin, it provokes no adverse irritation, can be removed on demand without pain, and leaves no tissue residue. Collectively, the hydrogel adheres to diverse substrates through non-covalent interactions at the interface combined with an effective energy dissipation mechanism within the network.

#### 2.2.5. Rheological Properties

**Figure 4:**
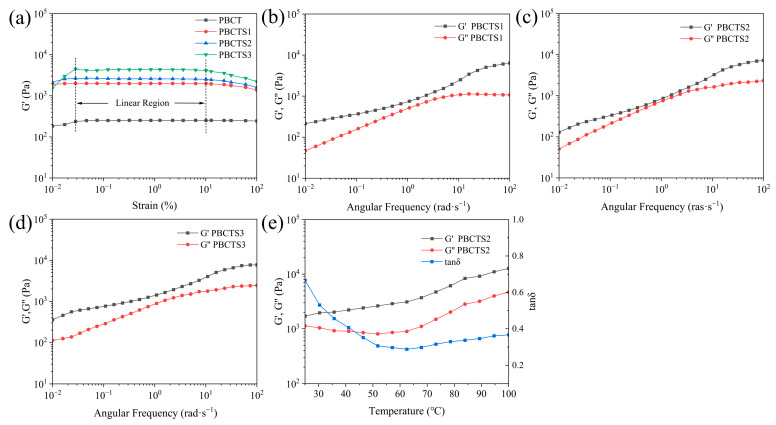
Rheological properties of hydrogels. (**a**) Determination of the LVR for PBCT, PBCTS1, PBCTS2 and PBCTS3 hydrogels. Angular frequency dependence of the storage modulus (G′) and the loss modulus (G″) for PBCTS1 (**b**), PBCTS2 (**c**), and PBCTS3 (**d**) hydrogels. (**e**) Temperature dependence of PBCTS2 hydrogels.

The rheological behavior of the hydrogels was probed using a rotational rheometer to elucidate the effect of SF on their viscoelasticity. Dynamic strain-sweep tests were first conducted to delineate the LVR. As shown in Figure 4a, the LVR spans 0.03–10% strain, within which G′ of all hydrogels remained constant and independent of the applied strain. A broader LVR generally indicates a denser and more uniform network, endowing the hydrogel with superior structural stability and greater resistance to mechanical stress [50,51]. SF addition enhances the mechanical properties and viscoelasticity of hydrogels through multiple mechanisms. During gelation, SF transitions from random coil to β-sheet, introducing additional crosslinking points that enhance network stability and integrity. Moreover, SF increases solution viscosity, reducing interchain distances and establishing the spatial proximity required for subsequent physical crosslinking via hydrogen bonding and other interactions.

Dynamic frequency sweeps were then performed at 1% strain within the linear viscoelastic region (LVR) to investigate the angular frequency dependence of PBCTS1, PBCTS2, and PBCTS3 hydrogels (Figure 4b–d). Both G′ and G″ of all hydrogels exhibited angular frequency dependence, with G′ consistently exceeding G″, indicating that the hydrogels form a stable elastic system [52,53].

PBCTS2 was selected for dynamic temperature scanning (Figure 4e). G′ remained consistently higher than G″ throughout the sweep, indicating that the elastic network remained intact and dominant. Both G′ and G″ increased with rising temperature, signifying thermally induced structural reorganization. Elevated temperature lowers solution viscosity, enhances polymer chain mobility, and allows kinetically hindered hydrogen bonding sites to reorganize into more stable crosslinks. For instance, heating a PVA–borax system accelerates the dynamic exchange and reorganization of hydroxyl–borate ester bonds [54]. Additionally, SF rapidly converts from random coil to β-sheet conformation during heating, increasing network rigidity [55].

#### 2.2.6. Self-Healing Tests

**Figure 5:**
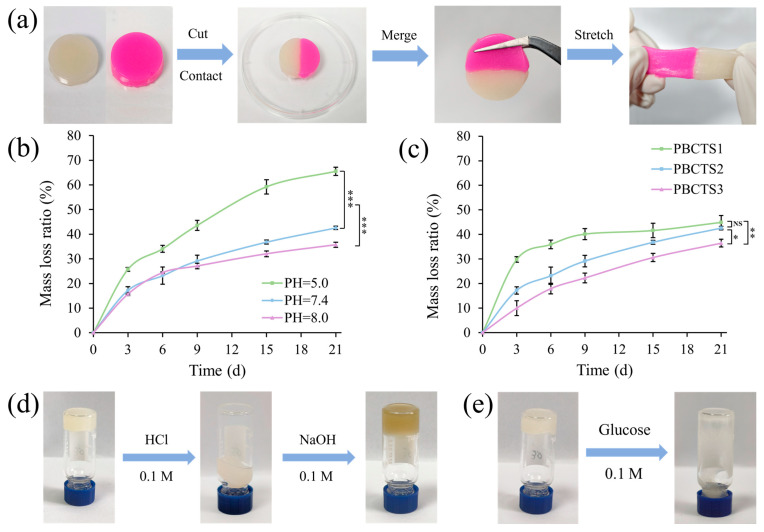
(**a**) Rapid self-healing phenomenon of PBCTS hydrogels stained with different colors. (**b**) Degradation profiles of PBCTS2 hydrogels in PBS solutions at various pH values. (**c**) Degradation profiles of hydrogels with different SF contents in PBS at a pH of 7.4. The pH-responsive behavior (**d**) and sugar-responsive behavior (**e**) of PBCTS2 hydrogels. NS *p* > 0.05, * *p* < 0.05, ** *p* < 0.01, *** *p* < 0.001.

The self-healing capability of PBCTS hydrogels was assessed via macroscopic tests. As shown in Figure 5a, severed hydrogels rapidly heal into an intact piece without external intervention, remaining stretchable with no visible pores at the interface. This rapid self-healing confirms a dynamic cross-linked network, primarily attributed to borate ester bonds (among PVA, TA, and borax) and hydrogen bonds (between SF and TA). Hydrogen bonds enable fast but weak reversible cross-linking, while borate ester bonds provide durable strength and water resistance. The synergy of both endows PBCTS hydrogels with rapid and robust self-healing.

#### 2.2.7. In Vitro Degradability

An ideal dressing should degrade appropriately after hemostasis and wound healing, avoiding long-term retention that may cause foreign-body reactions. Wound healing involves dynamic pH changes. Acute wounds typically exhibit a pH of 5.0–7.4, while infected or chronic wounds maintain a persistent pH above 7.5 [56,57]. We therefore investigated the in vitro degradation of the hydrogel in PBS buffers at pH values of 5.0, 7.4, and 8.0 (Figure 5b). Over time, all hydrogels degraded markedly. As the concentration of cleavable bonds decreased exponentially with hydrolysis, the degradation rate gradually declined. By day 21, degradation rates were 35.71%, 42.57%, and 65.51%, respectively, with significantly faster degradation at lower pH. Specifically, at pH < 6, borate ester bonds hydrolyze readily, leading to rapid degradation. Within the pH range of 7–9, however, borate exists as B(OH)_4_^−^ and forms stable tetrahedral complexes with diols, making the bonds less prone to hydrolysis and thus slowing degradation. We further evaluated the in vitro degradation of hydrogels with varying SF contents at a pH of 7.4 (Figure 5c). On day 21, degradation rates were 44.94%, 42.57%, and 36.47%, respectively. Mechanistically, higher SF contents increase cross-linking density and network strength, hindering solvent penetration and cross-link cleavage, thereby reducing the degradation rate.

#### 2.2.8. pH- and Sugar-Responsive Behavior

As shown in Figure 5d, adding 0.1 M HCl to the PBCTS hydrogel cleaves borate ester bonds in the acidic environment, yielding boric acid (B–OH) and polymer-side-chain hydroxyl groups (C–OH). The resulting drop in cross-link density causes the network to lose mechanical integrity and transition to a sol state. Subsequent addition of 0.1 M NaOH re-establishes borate ester bonds, returning the hydrogel to a gel state. The gelation mechanism primarily involves chemical cross-linking between PVA and borax. Similarly, adding glucose solution transforms the hydrogel into a sol state (Figure 5e). Glucose, rich in cis-diol structures, competes with PVA and forms a more stable borate–sugar complex, disrupting the original borate ester bonds. This causes the hydrogel to soften, swell, and completely dissociate into a sol. Thus, the PBCTS hydrogel exhibits dual pH- and sugar-responsive behavior, enabling environment recognition and on-demand action for applications in oral drug delivery, chronic wound management, and intelligent sensing.

### 2.3. In Vitro Evaluation of the PBCTS@PNF Hydrogels

#### 2.3.1. Drug Release Studies

**Figure 6:**
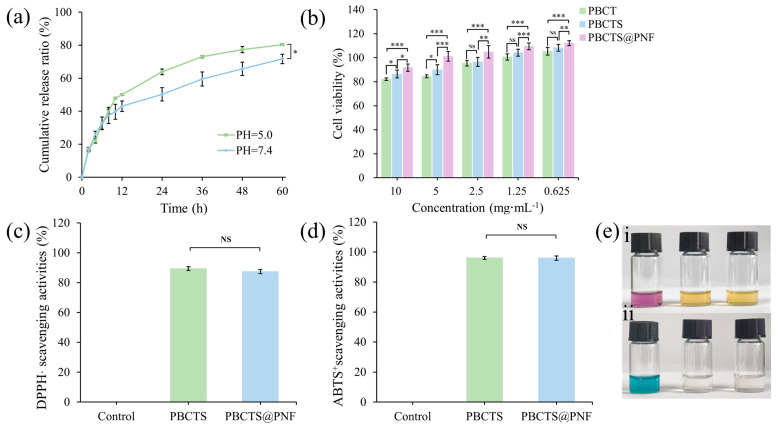
(**a**) In vitro release profiles of PNS-loaded hydrogels in media solution at a pH of 5.0 and pH of 7.4. (**b**) CCK-8 assay for cytocompatibility evaluation. DPPH⋅ (**c**) and ABTS·^+^ (**d**) free radical scavenging activities of PBCTS and PBCTS@PNF hydrogels. (**e**) Photographs showing the color change of DPPH⋅ (**i**) and ABTS⋅^+^ (**ii**) assays. (**e**) NS *p* > 0.05, * *p* < 0.05, ** *p* < 0.01, *** *p* < 0.001.

The cumulative release profiles of the PBCTS@PNF hydrogel in media of different pH solutions are shown in Figure 6a. In all media, the encapsulated PNF exhibited sustained release due to the dense three-dimensional network acting as a physical barrier to drug diffusion. The cumulative in vitro release of PNF reached its maximum at a pH of 5.0, where protons catalyze borate ester hydrolysis under mildly acidic conditions, reducing drug diffusion resistance and accelerating release. The release profiles at a pH of 5 and a pH of 7.4 were fitted using the Ritger–Peppas model, yielding diffusion coefficients of approximately 0.342 and 0.430, respectively. Both values are below 0.45, indicating that the drug release mechanism follows Fickian diffusion, with PNF released primarily via diffusion.

#### 2.3.2. Cytocompatibility Evaluation

NIH-3T3 mouse fibroblasts, which have simple culture requirements, high sensitivity to surface microenvironments, a well-defined genetic background, and close relevance to tissue repair, are widely accepted as the gold-standard model for in vitro biocompatibility testing. Hydrogel extracts of varying concentrations were prepared and co-cultured with NIH-3T3 cells. As shown in the CCK-8 assay results in Figure 6b, cell viability exceeded 80% at all extract concentrations, with no evidence of cytotoxicity, demonstrating excellent cytocompatibility. The favorable biocompatibility of the PBCTS@PNF hydrogel extract corroborates the synergistic effect of SF and PNF in promoting cell proliferation, a critical prerequisite for hydrogel application in wound healing.

#### 2.3.3. Antioxidant Activity

Antioxidant capacity is crucial for wound care hydrogels because oxidative stress represents one of the core pathological mechanisms underlying the impaired healing of chronic wounds. Excessive reactive oxygen species (ROS) lead to cellular damage, extracellular matrix degradation, and persistent inflammation. Antioxidant hydrogels can effectively scavenge ROS and modulate redox balance, thereby promoting the transition of wounds from the inflammatory phase to the proliferative phase and accelerating the healing process. The in vitro antioxidant capacity was evaluated using DPPH· and ABTS^+^ radical scavenging assays. The PBCTS2 and PBCTS@PNF hydrogels exhibited DPPH· scavenging rates of 89.45 ± 1.30% and 87.39 ± 1.42%, respectively, with a color change from purple to yellow (Figure 6c,e(i)). Their ABTS^+^ scavenging rates were 96.07 ± 0.93% and 95.95 ± 1.46%, with the solution turning from blue to colorless (Figure 6d,e(ii)). In the PBCTS hydrogel, the catechol moieties of TA rapidly donated hydrogen atoms to radicals, while Tyr and Trp residues in SF supplied electrons under neutral-to-weakly alkaline conditions, generating phenoxyl radicals that synergized with TA. Compared with that of PBCTS, the antioxidant capacity of PBCTS@PNF remained virtually unchanged, likely due to the brief testing duration (30 min) limiting PNF release, as only 16.04% liberation was achieved after 2 h at a pH of 7.4 in drug release assays. Additionally, the inherently high antioxidant activity of PBCTS may have masked any incremental contribution from the encapsulated drug.

#### 2.3.4. Antibacterial Activity

**Figure 7:**
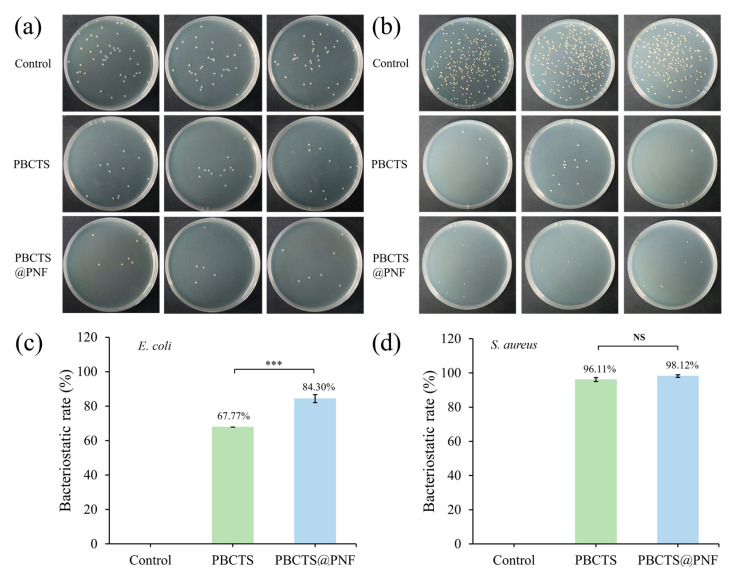
Photographs of the antibacterial efficacy tests against *S. aureus* (**a**) and *E. coli* (**b**). (**c**) Inhibition ratio of PBCTS hydrogels against *E. coli* and *S. aureus* at a dilution ratio of 1 × 10^7^. (**d**) Inhibition ratio of PBCTS@PNF hydrogels against *E. coli* and *S. aureus* at a dilution ratio of 1 × 10^5^. NS *p* > 0.05, *** *p* < 0.001.

Antibacterial performance is indispensable for wound-care hydrogels, as their moist, nutrient-rich matrix provides an ideal breeding ground for bacteria. Without built-in antimicrobial activity, planktonic bacteria rapidly form biofilms that sustain chronic inflammation and severely impede healing [58,59]. The antibacterial performance of the hydrogel was evaluated using the plate-count method with Gram-positive *Staphylococcus aureus (S. aureus)* and Gram-negative *Escherichia coli (E. coli)* as model strains (Figure 7). The PBCTS2 hydrogel exhibited inhibition rates of 67.77% against *E. coli* and 96.11% against *S. aureus*, while the PBCTS@PNF hydrogel showed superior activity with rates of 84.30% and 98.12%, respectively. The enhancement upon PNF incorporation was markedly more pronounced against *E. coli* (from 67.77% to 84.30%) than against *S. aureus* (from 96.11% to 98.12%, near-saturated). This differential effect can be explained by the distinct cell envelope structures of the two bacteria. *S. aureus* is a Gram-positive strain lacking an outer membrane, with a relatively accessible peptidoglycan layer; thus, PBCTS2 alone already achieves high efficacy (96.11%), leaving little room for further improvement. In contrast, *E. coli* is a Gram-negative bacterium possessing an additional outer membrane barrier that confers intrinsic resistance. The added PNF, whose antibacterial mechanism primarily involves disruption of bacterial cell membrane integrity via its amphiphilic saponin components, can effectively compromise the outer membrane of *E. coli* [60,61]. This disruption facilitates better penetration of the active components from PBCTS2, leading to a synergistic enhancement against the Gram-negative strain.

### 2.4. In Vivo Evaluation of the PBCTS@PNF Hydrogels

#### 2.4.1. PBCTS@PNF Accelerates Large-Area Acute Wound Healing

**Figure 8:**
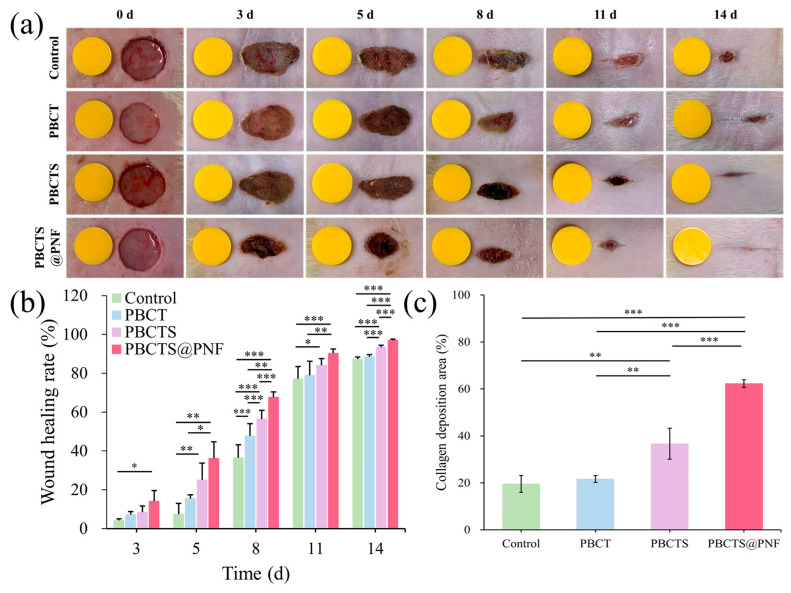
PBCTS@PNF hydrogel promotes the in vivo healing of acute wounds. (**a**) Representative photographs of the wound healing process in mice treated with control, PBCT, PBCTS, and PBCTS@PNF hydrogels over a 14-day period. (**b**) Quantification of in vivo wound healing rates on days 3, 5, 8, 11 and 14 (*n* = 6). (**c**) Quantitative analysis of collagen deposition on day 14 (*n* = 3). * *p* < 0.05, ** *p* < 0.01, *** *p* < 0.001.

Full-thickness skin defect models were established on the dorsal backs of SD rats to evaluate the therapeutic potential of PBCTS and PBCTS@PNF hydrogels in accelerating wound repair. Figure 8a,b show wound closure images for each group on days 0, 3, 5, 8, 11, and 14. Both PBCTS@PNF (96.97 ± 0.64%) and PBCTS (93.31 ± 1.12%) groups exhibited significantly faster healing rates than the control (87.31 ± 1.14%) and PBCT (88.50 ± 1.02%) groups (*p* < 0.05), along with markedly superior cosmetic appearance of regenerated skin. Notably, the PBCTS@PNF group achieved near-complete healing with negligible scarring by day 14. Collectively, these results demonstrate that the PBCTS@PNF hydrogel significantly accelerates wound healing.

#### 2.4.2. Histological Observation

**Figure 9:**
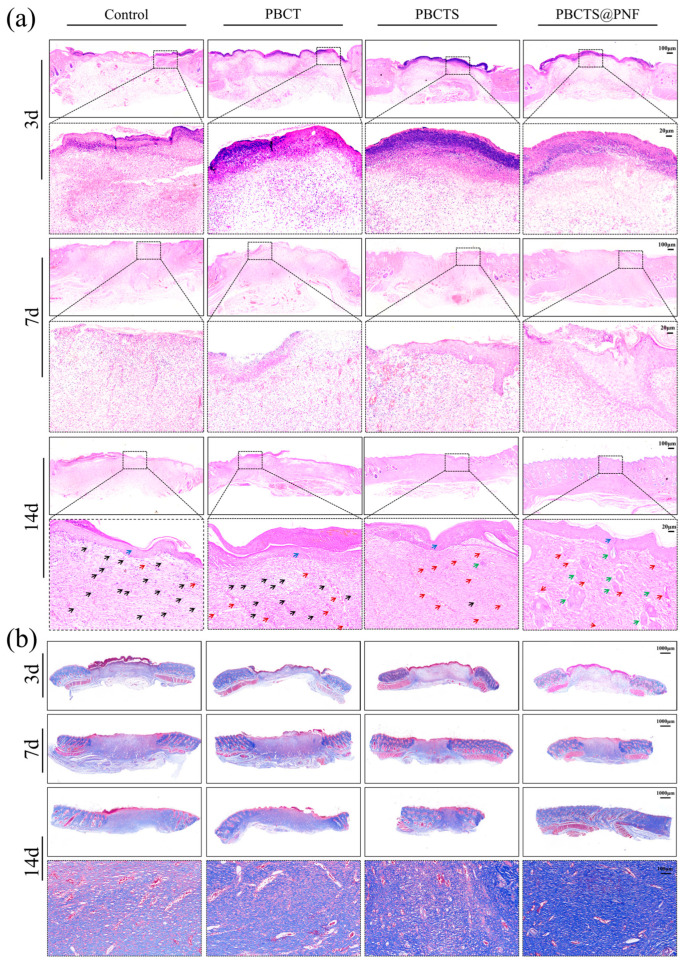
Representative images of H&E and Masson’s trichrome staining of tissues from each group. (**a**) H&E of wound tissues (black arrows indicate inflammatory cells; blue arrows indicate newly formed epidermis; red arrows indicate neovascularization; green arrows indicate hair follicles or sebaceous glands). (**b**) Masson staining of wound tissues. Histological sections from the central wound area, including the full-thickness wound bed (epidermis and dermis) and 2 mm margin of adjacent healthy tissue.

H&E staining showed the progressive stages of wound healing (Figure 9a). On day 3, all groups exhibited prominent inflammatory cell infiltration, characteristic of the early inflammatory phase. Notably, the control group showed particularly pronounced epidermal erosion and defect. In contrast, the PBCTS@PNF group displayed relatively milder inflammatory cell infiltration along with early granulation tissue formation, suggesting that PNF may accelerate the repair process by modulating the inflammatory response. On day 7, the control group displayed persistent extensive inflammation and negligible re-epithelialization. In the PBCT group, epidermal cell regeneration was observed, but a distinct epidermal layer was absent. The PBCTS2 group displayed relatively well-developed re-epithelialization. Notably, the PBCTS@PNF group exhibited significantly reduced inflammatory infiltration and superior re-epithelialization compared with the other three groups. By day 14, persistent inflammation was observed in partial wounds of the control and PBCT groups, whereas the PBCTS group showed substantially diminished inflammation with only scant residual inflammatory cells. Strikingly, complete elimination of inflammatory cells was achieved in the PBCTS@PNF group. All groups exhibited complete re-epithelialization and granulation tissue formation, albeit with variable maturity and density. Unlike the PBCTS group, the PBCTS@PNF group exhibited de novo hair follicle formation, densely organized granulation tissue, and abundant neogenic skin appendages, suggesting superior regenerative potential.

Masson staining revealed the progressive stages of wound healing (Figure 9b). During the early proliferative stage (day 7), nascent granulation tissue featured abundant red-stained fibroblasts and vascular structures, interspersed with sparse, disorganized blue collagen fibers. By the late remodeling phase (day 14), the PBCTS@PNF hydrogel group markedly accelerated the transition from hypercellular granulation tissue to mature collagen, exhibiting densely packed and organized collagen bundles that facilitated the restoration of normal dermal architecture. Quantitative collagen analysis by Masson staining further confirmed that PBCTS@PNF hydrogels promoted the transformation of hyperplastic granulation tissue into mature collagen (Figure 8c), facilitating the formation of a densely organized extracellular matrix while effectively preventing excessive fibrosis.

Histological examinations further corroborated the enhanced regenerative capacity of the PBCTS@PNF hydrogel. In the early stage of healing (day 3), the PNF released from the PBCTS@PNF hydrogel effectively modulated the wound inflammatory response. As healing progressed into the proliferative phase, the sustained release of PNF from the hydrogel further enhanced tissue regenerative capacity, as evidenced by promoted re-epithelialization and collagen maturation. H&E and Masson’s trichrome staining revealed markedly improved re-epithelialization, robust neovascularization, enhanced adnexal structure formation (including hair follicles), and increased collagen fiber proliferation in the PBCTS@PNF group.

## 3. Conclusions

Based on a dual dynamic crosslinking strategy, we successfully developed a multifunctional hydrogel (PBCTS@PNF). A key feature of this system is its ability to undergo self-gelation via physical crosslinking without the introduction of any chemical crosslinkers, thereby eliminating the biosafety risks associated with residual crosslinking agents. Compared to previously reported binary systems, such as the SF/TA system, which offers only single adhesive functionality, the CS/TA system, which lacks self-healing ability, or the PVA/borax system, which suffers from insufficient tissue adhesion, the PBCTS@PNF hydrogel integrates SF, TA, and CS and dynamic borate ester bonds into a single platform to achieve synergistic multifunctionality including rapid self-healing, on-demand removable adaptive adhesion, and pH/glucose dual-responsive sustained drug release, as well as excellent antibacterial, antioxidant, and pro-regenerative bioactivities. In a large-scale acute wound model, PBCTS@PNF modulated the wound microenvironment to alleviate inflammation and markedly promote re-epithelialization, angiogenesis, hair follicle neogenesis, and orderly collagen deposition, thereby accelerating wound closure. In summary, the PBCTS@PNF multifunctional hydrogel integrates antioxidant, antibacterial, and pro-healing functions, overcoming the limitations of single-function traditional dressings. The established controlled release system combining a hydrogel carrier with active ingredients of Traditional Chinese Medicine achieves synergistic pro-healing effects of materials and drugs, offering a highly promising therapeutic platform with extensive applicability in tissue regeneration.

## 4. Materials and Methods

### 4.1. Materials

The total saponins of *Panax notoginseng* flower buds (PNF) were extracted in the laboratory (saponins purity > 86%, UV). Bombyx mori silk was purchased from Yizhou, Guangxi. Sodium carbonate (Na_2_CO_3_, ≥99.8%), poly(vinyl alcohol) 1799 (PVA, alcoholysis degree: 98–99%), borax (sodium tetraborate decahydrate, Na_2_B_4_O_7_·10H_2_O, ≥99.5%), calcium chloride anhydrous (CaCl_2_, ≥96%) and chitosan (CS, viscosity: 50–800 mPa·s) were obtained from Sinopharm Chemical Reagent Co., Ltd. (Shanghai, China). Tannic acid (TA, ≥99.0%) was acquired from Aladdin Industrial Corporation Co., Ltd. (Shanghai, China). CCK-8 was supplied from GlpBio (Shanghai, China). Fetal bovine serum was acquired from Bioagrio (Shanghai, China). Dulbecco’s modified eagle medium (DMEM) with high glucose was purchased from MeilunBio (Shanghai, China). All other reagents were commercially purchased and used without further purification, meeting analytical grade standards. Sprague–Dawley (SD) rats (*n* = 48, male with a body weight of 200–220 g) were obtained from Bikai Keyi Biotechnology Co., Ltd. (Shanghai, China) and used to investigate the wound healing-promoting effect of the hydrogel in this study. The study protocol has been reviewed and approved by the Laboratory Animal Welfare and Ethics Committee of Shanghai University of Traditional Chinese Medicine (PZSHUTCM2509050013). All chemicals were of analytical grade and used without further purification.

### 4.2. Preparation of SF Solution

*Bombyx. mori* silk was maintained at 100 °C in 0.05% Na_2_CO_3_ solution for 30 min, and then rinsed with deionized water. The procedure was duplicated twice and then dried to harvest the degummed silk. The degummed silk was slowly dissolved into CaCl_2_/C_2_H_5_OH/H_2_O (1:2:8) solution at 60 °C, transferred to a dialysis bag, and then dialyzed against deionized water for 72 h. After lyophilization, SF was obtained.

### 4.3. Preparation of the Hydrogels

PVA powder was dissolved in deionized water under magnetic stirring (90 °C) for 2 h to form a homogeneous solution. Subsequently, CS solution and SF solution were added to the PVA solution, followed by stirring for 1 h to yield Solution A. TA powder and borax were dissolved in deionized water to prepare Solution B. Solution B was then slowly added to Solution A under stirring to form a uniform hydrogel. Finally, the resulting hydrogel was frozen at −20 °C for 2 h and subsequently thawed at room temperature to obtain PBCTS hydrogel. The PBCTS hydrogel was composed of: PVA—9.0%; borax—0.06%; CS—0.01%; and TA—2.0%. The hydrogels with SF contents of 1.5%, 3.0%, and 4.5% were respectively named PBCTS1, PBCTS2, and PBCTS3. After adding 1.0 g of PNF to Solution A and mixing thoroughly, Solution B was introduced dropwise. The remaining steps were identical to those used for PBCTS2 hydrogel preparation, yielding the PBCTS@PNF hydrogel.

### 4.4. Characterization of the PBCTS Hydrogel

#### 4.4.1. Structural Characterization

The chemical bonds and functional groups of the hydrogel samples were determined by recording the infrared spectra using Fourier transform infrared spectroscopy (FTIR, Nicolet Summit X, Waltham, MA, USA) with the attenuated total reflection (ATR) technique. The measurement wavelength range for all samples was from 4000 cm^−1^ to 400 cm^−1^, with 32 scans and a resolution of 4 cm^−1^. The crystalline behavior of the hydrogel samples was analyzed employing X-ray diffraction (XRD, Ultimate IV, Akishima, Japan). The patterns were obtained under the conditions of a scanning rate of 2°·min^−1^, an accelerating voltage of 35 kV, and a current of 10 mA, with the diffraction angle ranging from 10° to 80°. The microstructure of the hydrogels was investigated via scanning electron microscopy (SEM, Zeiss Sigma 360, Oberkochen, Germany). After freeze-drying, the samples were rapidly fractured in liquid nitrogen to expose their internal structure, followed by gold sputtering to enhance the contrast for SEM imaging. The porosity of the hydrogels was determined through the liquid displacement method [62]. The hydrogels were freeze-dried and weighed (M_0_), and then immersed in anhydrous ethanol until fully saturated, after which they were weighed again (M_e_). The porosity was calculated according to the following formula:

$$\mathrm{Porosity}\;(\%)\;=\;\frac{\left(M_{e}-M_{0}\right)}{\rho V}\times 100\%$$

where ρ is the density of anhydrous ethanol and V is the volume of the freeze-dried hydrogels.

#### 4.4.2. Water Retention Properties

Glycerol was added to enhance the moisture retention of the hydrogels. The hydrogels were shaped into cylinders of identical volume, and their initial mass (M_0_) was recorded. The hydrogels were placed at room temperature for 60 h, with their mass recorded at different time points (M_t_). The water retention ratio was calculated as follows:

$$\mathrm{Water}\;\mathrm{retention}\;\mathrm{ratio}\;(\%)\;=\;\frac{M_{t}}{M_{0}}\times 100\%$$

#### 4.4.3. Swelling Properties

The swelling properties of the hydrogel under physiological conditions were determined gravimetrically using a weight-based method. Freeze-dried hydrogels (M_0_) were fully immersed in 10 mM PBS buffer (pH of 7.4) and incubated at 37 °C with orbital shaking at 100 r·min^−1^ until equilibrium swelling was achieved. Excess surface water was carefully removed with filter paper, and the sample was immediately weighed (Mt). The swelling ratio was calculated according to the following formula:

$$\mathrm{Swelling}\;\mathrm{ratio}\;(\%)\;=\;\frac{\left(M_{t}-M_{0}\right)}{M_{0}}\times 100\%$$

#### 4.4.4. Adhesive Properties

To evaluate the adhesive strength of the hydrogel toward dissimilar substrates, lap-shear tests on porcine skin and polytetrafluoroethylene (PTFE) were performed with an electronic universal testing machine (HDW-5, Jinan Hengxu, China). The adhesive strength of the hydrogels was calculated according to the following formula:

$$\mathrm{Adhesive}\;\mathrm{strength}\;(\mathrm{kPa})\;=\;\frac{F}{A}$$

where F (N) is the maximum tensile load and A (m^2^) is the overlapped area of the two adherends.

#### 4.4.5. Rheological Properties

The samples were prepared into a cylindrical shape (diameter = 20 mm). The rheological behaviors of hydrogels were analyzed using a rotary rheometer (MARS 60, USA). Dynamic strain scans (γ = 0.01–100%) were conducted at 25 °C and a constant angular frequency of 6.28 rad·s^−1^ to delineate the linear viscoelastic region (LVR). Dynamic frequency scans (ω = 0.01–100 rad·s^−1^) were performed at 25 °C under a fixed strain of γ = 1% to record G′ and G″ moduli of the hydrogels. Dynamic temperature scans (T = 25–100 °C) were performed at γ = 1% and ω = 6.28 rad·s^−1^, recording the temperature-dependent G′ and G″ moduli. The loss tangent (tanδ) was calculated as follows:

$$\tan\;\delta =\frac{G^{\prime \prime }}{G^{\prime }}$$

#### 4.4.6. Self-Healing Tests

Without applying any external force, the unstained and Rhodamine B-stained PBCTS hydrogels were simply placed in contact, and their self-healing behavior was observed macroscopically.

#### 4.4.7. In Vitro Degradability

In vitro degradation was quantified gravimetrically by immersing pre-weighed hydrogels (M_0_) in PBS buffers of pH values of 5.0 and 7.4 at 37 °C under 100 r·min^−1^ orbital shaking. At predetermined intervals, samples were retrieved, their surface moisture was gently removed with filter paper, and they were weighed (M_t_). The in vitro degradation ratio was calculated according to the following formula:

$$\mathrm{Degradation}\;\mathrm{ratio}\;(\%)\;=\;\frac{\left(M_{0}-M_{t}\right)}{M_{0}}\times 100\%$$

#### 4.4.8. pH- and Sugar-Responsive Behaviors

The pH- and saccharide-responsiveness of the PBCTS hydrogel was investigated by macroscopically observing the sol–gel transition. HCl solution (0.1 M) was added to the vial containing the PBCTS hydrogel; the vial was then inverted and the gel state was observed. After the gel disintegrated, NaOH solution (0.1 M) was introduced into the same vial. An aliquot of high-concentration glucose solution (10 mg·mL^−1^) was added to the vial containing the hydrogel, and the vial was inverted and monitored for gel disintegration.

### 4.5. In Vitro Evaluation of the PBCTS@PNF Hydrogels

#### 4.5.1. Drug Release Studies

The drug release of the PBCTS@PNF hydrogels was evaluated in PBS buffers at pH values of 5.0 and 7.4. An appropriate amount of PBCTS@PNF hydrogel was placed in 10 mL of PBS buffer and incubated in a shaking bath (37 °C, 100 r·min^−1^). At selected time intervals, 1 mL of the extraction medium was withdrawn and replaced with an equal volume of fresh PBS. The absorbance was measured at 548 nm by UV–Vis spectrophotometry.

#### 4.5.2. Cytocompatibility Evaluation

A CCK-8 assay was employed to evaluate the in vitro biocompatibility of the hydrogels. Irradiation-sterilized hydrogel specimens were soaked in DMEM supplemented with 10% fetal bovine serum and 1% penicillin / streptomycin at 37 °C for 24 h to obtain a series of extracts. NIH-3T3 mouse fibroblasts were seeded into 96-well plates at a density of 2 × 10^4^ cells per well and cultured for 24 h in a 5% CO_2_ humidified incubator at 37 °C. The old medium was then discarded, and the cells were further co-cultured with the hydrogel extracts for another 24 h. Finally, the extracts were discarded, 10 μL of CCK-8 solution was added, and the plates were incubated in the dark for the designated period. The optical density (OD) was measured at 450 nm using a microplate reader. The cell viability was calculated according to the following formula:

$$\mathrm{Cell}\;\mathrm{viability}\;(\%)\;=\;\frac{\left({\mathrm{OD}}_{s}-{\mathrm{OD}}_{b}\right)}{\left({\mathrm{OD}}_{c}-{\mathrm{OD}}_{b}\right)}\times 100\%$$

where OD_s_, OD_b_, and OD_c_ represent the absorbances of the sample wells, blank wells, and negative-control wells, respectively.

#### 4.5.3. Antioxidant Activity

Sixty milligrams of hydrogel sample were immersed in 3 mL of the DPPH·/ethanol solution and stored in the dark at room temperature for 30 min. The absorbance (Ab_s_) at 517 nm was then recorded using UV spectrophotometer. Sixty milligrams of hydrogel sample were weighed and immersed in 3 mL of pre-prepared ABTS^+^ solution and then incubated at room temperature in the dark for 30 min. Abs at 734 nm was measured using a UV spectrophotometer. The DPPH· and ABTS^+^ scavenging activities were calculated as follows:

$$\mathrm{Scavenging}\;\mathrm{activity}\;(\%)\;=\;\frac{\left({\mathrm{Abs}}_{b}-{\mathrm{Abs}}_{s}\right)}{{\mathrm{Abs}}_{b}}\times 100\%$$

where Abs_b_ is the absorbance of the fresh DPPH·/ethanol or ABTS^+^ solution and Abs_s_ is the absorbance of the mixture after the hydrogel has been incubated in the DPPH·/ethanol or ABTS^+^ solution.

#### 4.5.4. Antibacterial Activity

The antibacterial activity of the hydrogel was evaluated by determining the inhibition rates against *E. coli* and *S. aureus*. An aliquot of 0.5 mL bacterial suspension (10^6^ CFU·mL^−1^) was added to a sterile culture tube containing the hydrogel, followed by static incubation at 37 °C for 24 h. After incubation, the bacterial suspension was subjected to serial 10-fold dilutions in sterile PBS. Then, 100 μL of each dilution was evenly spread on LB agar plates and incubated at 37 °C for 18 h.

### 4.6. In Vivo Evaluation of the PBCTS@PNF Hydrogels

#### 4.6.1. Establishment of a Full-Thickness Skin Defect Model

Forty-eight Sprague Dawley (SD) rats were randomly divided into four groups, which consisted of the blank control group, the PBCT hydrogel group, the PBCTS hydrogel group, and the PBCTS@PNF hydrogel group. After the acclimatization period, the rats were shaved on the dorsum using an electric clipper one day before wound creation (3 cm × 3 cm). The following day, under isoflurane anesthesia, circular full-thickness skin defects (1.2 cm in diameter) were created on the dorsum using sterile scissors and forceps. Hydrogels were replaced daily.

#### 4.6.2. Wound Healing Ratio

Wound size was documented photographically with a 1 cm scale bar. The wound area was calculated with AutoCAD 2020 software to determine the wound healing ratio. The wound healing ratio was calculated according to the following formula:

$$\mathrm{Wound}\;\mathrm{healing}\;\mathrm{ratio}\;(\%)\;=\;\frac{\left(A_{0}-A_{t}\right)}{A_{0}}\;\times 100\%$$

where A_0_ is the original wound area at day 0 and A_t_ is the wound area at different times.

#### 4.6.3. Histological Observation

Full-thickness skin specimens harvested on days 3, 7, and 14 were used to prepare histological sections taken from the central portion of each wound, spanning the entire wound bed including the epidermis, dermis, and a 2 mm margin of surrounding healthy tissue. These sections were then subjected to H&E and Masson staining.

### 4.7. Statistical Analysis

All experiments were performed at least three times (*n* ≥ 3). Statistical analysis was performed using IBM SPSS Statistics 27.0 (SPSS Inc., Chicago, IL, USA) with one-way ANOVA. Values of * *p* < 0.05, ** *p* < 0.01 and *** *p* < 0.001 were considered to indicate statistically significant differences between groups. When *p* ≥ 0.05, differences among groups were regarded as not statistically significant (NS).

## Data Availability

The data presented in this study are available upon request from the corresponding author.
